# Risk-adjusted capitation payment for outpatient services in China's healthcare insurance: a case study of an affluent city in southern China

**DOI:** 10.3389/fpubh.2026.1774676

**Published:** 2026-03-13

**Authors:** Sun Jinnan, He Qiong, Liu Ke, Du Wenwen, Xu Wei

**Affiliations:** 1 School of International Pharmaceutical Business, China Pharmaceutical University, Nanjing, China; 2 Key Laboratory of Systems Biomedicine(Ministry of Education), Shanghai Center for Systems Biomedicine, Shanghai Jiao Tong University, Shanghai, China

**Keywords:** capitation, healthcare insurance, outpatient, reform of healthcare insurance payment, risk-adjusted

## Abstract

**Introduction:**

This study explores the feasibility of a risk-adjusted outpatient capitation payment model for China's Urban Employee Basic Healthcare Insurance (UEBHI) and Urban-Rural Resident Basic Healthcare Insurance (URRBHI), using 2023–2024 medical insurance settlement data from GZ, an economically developed city in southern China.

**Methods:**

Through multiple linear regression analysis, gender, age, Class A Outpatient Chronic and Special Diseases (OCSDs) status, and inpatient status were identified as key risk-adjustment factors (*p* < 0.001), while length of hospital stay was excluded due to its lack of statistical significance in the UEBHI model (*p* = 0.917). K-means clustering was applied to optimize groupings: age was categorized into 3 groups for both insurance schemes, and single Class A OCSDs were divided into 4 groups (for UEBHI) and 5 groups (for URRBHI), with low intra-group coefficients of variation (CV) ensuring grouping precision.

**Results:**

A multi-year weighted adjustment mechanism was introduced to smooth fluctuations from single-year data. When equal weights were assigned to 2023 and 2024 data, the model yielded a simulated municipal healthcare insurance fund saving potential of 641 million RMB, accounting for 5.47% of the actual total outpatient fund expenditure in 2024. It should be clarified that this saving potential is a model-based estimate derived from the simulation, not an observed outcome after real-world reform implementation.

**Discussion:**

This study provides a referenceable technical framework for China's outpatient healthcare insurance payment reform in economically developed regions of China, while acknowledging limitations such as the single-case sample (GZ only) and the absence of socioeconomic and lifestyle factors in the model. Future research could further refine the model by incorporating additional influencing factors and expanding the sample scope.

## 1 Introduction

The healthcare security system is a crucial component of the social security system, and the reform of its payment methods plays a central role in achieving universal health coverage, controlling medical costs, and improving the quality and efficiency of healthcare services. For a long time, the fee-for-service (FFS) payment model, while stimulating the supply of medical services and meeting the diverse needs of patients, has also led to issues such as over-treatment and unreasonable growth in medical expenses. These problems have severely undermined the sustainability of healthcare funds and compromised the fairness and accessibility of medical services (1, 2). In response to these challenges, countries around the world are actively exploring and implementing diversified reforms in healthcare payment methods. The aim is to fundamentally alter the behavioral incentives for healthcare providers, strike a balance between the volume and quality of medical services, and thereby promote the sustainable development of the healthcare system.

In China, the reform of healthcare payment methods constitutes a core component of deepening the healthcare system reform. Since the launch of the new round of healthcare reform in 2009, the national level has continuously advanced payment method reforms, shifting from the traditional fee-for-service model toward composite payment methods such as diagnosis-related groups (DRG) and diagnosis-intervention packet (DIP) payments, capitation, and global budget payments. This transition aims to establish a more scientific and rational healthcare payment system (1). While the early focus of reform was primarily on inpatient services, as seen in the piloting and scaling of DRG and DIP, the reform of outpatient payment methods has gained increasing attention within the context of the coordinated “tripartite reform” of healthcare, pharmaceuticals, and medical insurance. Outpatient services are characterized by high volume, diverse and complex case types, and fragmented cost structures, presenting unique practical challenges for payment reform.

In September 2021, the General Office of the State Council issued the *14th Five-Year Plan for National Healthcare Security*, a key guiding policy for China's healthcare security development. The plan sets the important goal of continuously deepening healthcare payment method reforms. It outlines specific requirements, including “formulating technical specifications for total healthcare fund budget management, per diem payments, and capitation payments”; “improving healthcare payment policies for tightly integrated medical consortia”; and “deepening outpatient payment method reforms, standardizing basic outpatient payment units, and gradually establishing payment methods based on service capacity, service items, and service volume to guide appropriate care-seeking and promote primary care first-contact”. For instance, regarding DRG, the plan emphasizes promoting the pilot experience of DRG nationwide and continuously optimizing the grouping methodology. Regarding capitation payments, it advocates for integrating capitation for general outpatient services at primary healthcare institutions with family doctor contract services, implementing capitation for chronic diseases with clear diagnosis and treatment protocols and assessment indicators—such as diabetes, hypertension, and chronic renal failure—and strengthening chronic disease management.

In addition to the *14th Five-Year Plan for National Healthcare Security*, the Chinese government has introduced numerous other policies to guide the reform of outpatient medical insurance payment methods. As early as 2017, *the Guiding Opinions of the General Office of the State Council on Further Deepening the Reform of Basic Medical Insurance Payment Methods* proposed that for primary healthcare services, capitation payment may be adopted, and exploration should be made to combine capitation with chronic disease management; for complex cases and outpatient expenses not suitable for bundled payment, FFS may be applied. In 2020, *the Opinions of the Central Committee of the Communist Party of China and the State Council on Deepening the Reform of the Medical Security System* called for “gradually incorporating outpatient medical expenses into the payment scope of the basic medical insurance pooling fund, reforming the individual accounts of employee basic medical insurance, and establishing a sound outpatient mutual-aid mechanism”. This document also reiterated the adoption of capitation payment for specific chronic outpatient conditions. In 2021, *the General Office of the State Council issued the Guiding Opinions on Establishing and Improving the Outpatient Mutual-Aid Mechanism for Employee Basic Medical Insurance*, which proposed measures such as “lowering the outpatient deductible threshold, raising the maximum payment limit, and adjusting the method of individual account contributions”, as well as “including outpatient expenses for certain diseases with long treatment cycles, significant health impacts, and high cost burdens into the coverage scope for chronic outpatient conditions”. The introduction of these numerous policy documents has provided directional guidance for the ongoing reform of outpatient payment methods.

In long-term international practice, various outpatient payment models beyond FFS have gradually been developed, such as capitation, regional global budgets, and Ambulatory Patient Groups (APG). However, capitation may lead to patient dumping or selective provision of services by healthcare providers (3–5). Regional global budgets, if inappropriately set, may cause healthcare institutions to reduce necessary services to control costs (6). The APG pilot implemented in Jinhua, Zhejiang, relies heavily on high-quality data and information systems for effective execution and is susceptible to moral hazards such as splitting patient visits to obtain unreasonable insurance reimbursements (7). Considering these challenges, this study takes an affluent city in southern China as a case study to explore the feasibility of introducing risk-adjustment factors into the traditional flat-rate capitation model. The aim is to enhance the efficiency of healthcare fund payments and mitigate potential issues such as patient dumping and selective service provision.

Prior capitation payment studies in China are either limited to single disease populations or single insurance schemes, and few have integrated inpatient status as a risk-adjustment factor or developed a multi-year weighted adjustment mechanism for outpatient capitation. This study fills these gaps by constructing a risk-adjusted capitation model for the general population covering both UEBHI and URRBHI, incorporating inpatient status as a novel risk factor, and designing a multi-year weighted mechanism to smooth single-year data fluctuations.

## 2 Literature review

### 2.1 Inpatient payment reform in China

Extensive practice in China has demonstrated that the DRG/DIP payment reforms can effectively reduce inpatient medical costs (8, 9, 27, 28), decrease length of hospital stay, and lower overall healthcare fund expenditures (10, 11). However, some studies point to complexities and potential adverse effects of DRG/DIP in cost containment. Empirical evidence from multiple regions in China indicates that while these payment reforms have led to an overall reduction in healthcare fund spending, total hospitalization expenses for patients have not decreased. Instead, they have risen, primarily driven by an increase in out-of-pocket payments by patients (12–14). The moral hazard behavior of healthcare institutions induced by such payment reforms is likely to also manifest in the context of outpatient payment system reforms. Numerous studies indicate that DRG/DIP reforms may incentivize hospitals to shift certain services or costs from inpatient to outpatient settings to control hospitalization expenses, resulting in a phenomenon known as “cost shifting” (12, 13). Therefore, as two integral components of healthcare delivery, any discussion on reforming outpatient payment methods cannot be separated from an examination of inpatient payment system reforms.

### 2.2 Outpatient payment reform

#### 2.2.1 Capitation all over the world

As a relatively mature payment method, capitation has been widely implemented across the globe. However, precisely because provider revenue under this model is constrained by both the number of enrolled patients and treatment costs, a flat-rate capitation system inherently incentivizes healthcare providers to engage in behaviors such as under-provision of services, excessive referrals, and adverse selection of healthier or less complex patients (15). For instance, study on capitation for outpatient services in primary care institutions in Ghana found a decline in outpatient service utilization following its implementation (16, 17). Research in the United States also indicates that after introducing capitation for primary care physicians, patients experienced increased hospitalizations alongside a corresponding decrease in outpatient visits (18). In Ontario, Canada, a study on a mixed-payment model incorporating capitation similarly showed a reduction in services provided by primary care physicians after its adoption (19). In Indonesia, a pilot payment model combining capitation with performance-based elements, initiated in 2015, demonstrated no significant change in referral rates for conditions not requiring specialized care, suggesting the policy effect fell short of expectations (20). Another study exploring the hypothetical adjustment of capitation rates based on patient severity and regional factors found that such adjustments may not necessarily lead to fairer access to healthcare services (21). These studies collectively suggest that if outpatient capitation standards are not set precisely, they may fail to achieve the intended regulation of medical behaviors.

However, other research has drawn different conclusions. A study in the Taiwan (a region of PRC) found that while capitation under a global budget reduced overall outpatient volume, there was no significant change in the volume of services related to patient recovery or severe illnesses (22). An evaluation of the diabetes capitation policy in Tianjin, China, showed that after implementation, medical costs and complication rates for diabetic patients decreased, total outpatient visits declined significantly, and the proportion of visits to primary care institutions increased (23). Another study focusing on rural diabetic patients in China also noted that after the implementation of capitation, the average outpatient costs for patients with type 2 diabetes decreased, and prescribing practices at township health centers became more standardized (24). Furthermore, related research from Tianjin suggested that a blended payment model combining risk-adjusted capitation—based on factors such as age, gender, and complications (especially diabetes-related ones)—with fee-for-service payments might yield better outcomes (25). A modeling study based on data from 14 U.S. states indicated that a performance-linked capitation standard, adjusted for the health status of the local patient population, could potentially achieve socially optimal outcomes (29).

In summary, whether capitation can effectively control medical costs and guide provider and patient behavior remains inconclusive, varying across different policy contexts and target populations.

#### 2.2.2 Ambulatory patient groups

Implemented in Jinhua (Central city of Zhejiang Province), APG represent a bundled payment model for outpatient services, analogous to DRG used for inpatient care. A quasi-experimental study on this payment reform found that APG contributed to a reduction in outpatient fund expenditures and effectively promoted tiered healthcare delivery within the region. Similarly, an evaluation of the pilot outcomes in 2020 indicated that this reform helped establish a comprehensive governance mechanism for medical services, fostered the development of both healthcare insurance and public hospitals, optimized service quality, and improved fund efficiency (7).

### 2.3 Key mechanisms shaping outpatient payment reform

The design and evaluation of outpatient payment models are inherently driven by three core mechanisms that interact to influence the behavior of healthcare providers and the sustainability of healthcare insurance funds. Clarifying these mechanisms provides the theoretical underpinning for the risk-adjusted capitation model constructed in this study.

#### 2.3.1 Risk selection

Under a flat-rate capitation system, healthcare providers are reimbursed a fixed amount per enrollee, which is uncoupled from actual clinical costs. This creates a natural incentive for providers to cherry-pick healthier patients or those with less complex conditions—a practice that not only erodes the equity of healthcare access but also concentrates high-risk patients in specific facilities, ultimately jeopardizing the financial stability of health insurance funds. To mitigate this issue, this study integrates a comprehensive set of risk factors and employs precise risk stratification to calibrate differentiated capitation payment standards. This design is intended to attenuate providers' incentives for adverse selection and guarantee that institutions caring for high-risk patient populations are sufficiently compensated.

#### 2.3.2 Cost shifting

The advancement of DRG/DIP payment reforms for inpatient services has given rise to a “cost shifting” phenomenon, whereby healthcare institutions transfer certain medical costs or services from the inpatient to the outpatient setting to control hospitalization expenses. This cross-setting cost transfer not only distorts the structure of medical expenditures but also exacerbates the payment pressure on outpatient health insurance funds. Consequently, outpatient payment reform cannot be implemented in isolation and must be designed in coordination with the inpatient payment system. To address this, factors related to inpatient care are incorporated as core risk-adjustment variables, fully accounting for the characteristic that patients with a prior hospitalization history have higher outpatient service needs and associated costs.

#### 2.3.3 Moral hazard

Outpatient payment reforms may induce moral hazard behaviors among healthcare providers, including under-provision of necessary medical services, excessive referrals to higher-level medical institutions, or maximizing medical insurance reimbursement benefits through visit-splitting. These behaviors not only undermine the quality and efficiency of medical services but also result in the waste of healthcare insurance funds. The risk-adjusted capitation payment model intended to be constructed in this study aims to mitigate the risks. By scientifically setting capitation standards for different populations, it weakens healthcare providers' incentives to cut back on necessary services. No single system exists in isolation, and it is imperative to formulate matching assessment mechanisms during the actual implementation of the policy.

Therefore, the outpatient payment reform implemented alongside inpatient payment reform has indeed enhanced the efficiency of healthcare fund allocation and improved medical service behaviors. However, it is also evident that a single, simplistic payment model may no longer suffice to meet the evolving needs of contemporary society. There is a compelling need to explore the integration of multidimensional factors—such as disease profiles and demographic indicators—into the design of payment standards, thereby enabling more refined and precise healthcare fund allocation.

## 3 Data and methods

### 3.1 Original data

#### 3.1.1 Research sample

In terms of the selection of the sample city, China's economic development generally shows that eastern coastal provinces are ahead of the national average. Meanwhile, the overall medical care level in these provinces is also higher than that in most other regions of the country. Therefore, for conducting research on outpatient payment method reform, which is closely related to medical care level and economic development level. The study selected GZ, a leading economically developed city in southern China, as the research object based on the consideration of data availability and the maturity of medical insurance system construction in developed regions. China has significant regional disparities in healthcare resources, epidemiological profiles, insurance generosity, and provider incentives across different provinces (26), and the research context of economically developed cities is different from that of less-resourced regions. Thus, the research design of this study is oriented to providing a policy case reference for developed regions with similar medical insurance and medical service development levels. Additionally, since outpatient treatment is mostly targeted at patients who are permanent local residents, cross-regional medical service users are temporarily excluded from this model. GZ, as an economically developed southern city with sufficient healthcare insurance funds and high-quality medical resources, provides a valuable case for exploring the technical design of risk-adjusted capitation. However, the findings may overestimate the feasibility of this model in less-resourced regions with insufficient fund capacity and relatively weak primary medical care systems. Adaptations based on local fund status, disease spectrum, and medical resource allocation are required for broader application.

The outpatient pooling system of Urban Employee Basic Healthcare Insurance (UEBHI) was fully implemented nationwide by the end of 2022. Its direct impact on healthcare insurance funds is that the UEBHI fund expenditure has been significantly higher than in previous years since 2023. Additionally, as of 2025 when this study was conducted, the settlement of medical insurance funds for 2025 had not yet been completed (which is generally finalized in around April of the following year). Thus, 2023 and 2024 were selected as the study time frame.

While relatively well-defined clinical pathways have been established for inpatient care, outpatient diagnostic and treatment practices are considerably more complex. Furthermore, different regions across China maintain their own distinct lists of outpatient chronic and special diseases (OCSDs). Consequently, this study selected diseases included in the Class A OCSD list of the sample city as the disease spectrum for risk adjustment considerations. Meanwhile, in the city's prior policy implementations, capitation payment has been closely integrated with the family doctor contract system—a primary healthcare delivery model wherein individuals sign contracts with primary care facilities to access basic medical and health services. Therefore, diseases within the Class A OCSDs list that are not suitable for management in primary care settings were excluded, ultimately forming the final disease spectrum incorporated into this risk-adjustment model: epilepsy, systemic lupus erythematosus (SLE), bronchial asthma (BA), Parkinson's disease (PD), chronic renal insufficiency (non-dialysis) (CRI-ND), coronary heart disease (CHD), rheumatoid arthritis (RA), ulcerative colitis (UC), chronic glomerulonephritis (CG), Sequelae of Cerebrovascular Disease (SCD), diabetes mellitus (DM), hypertension, chronic obstructive pulmonary disease (COPD), osteoarthritis (OA), hyperlipidemia, chronic heart failure (CHF), Crohn's disease (CD), Liver Cirrhosis (LC), hypothyroidism, psoriasis, ankylosing spondylitis (AS).

#### 3.1.2 Data sources and variable definitions

In the sample city, the fund expenditures of UEBHI and URRBHI across medical institutions of different levels (Third-Level, Second-Level, and Primary Medical Institutions) exhibit the characteristics of non-normal distribution, heteroscedasticity, and a large sample size (*n* > 10,000). The statistical significance results of the Kolmogorov–Smirnov (K–S) test indicated that there were statistically significant differences between each pair of medical institution levels (*p* < 0.001), with a distinct decreasing trend from Third-Level to Primary Medical Institutions. Therefore, in the context of promoting primary care first consultation and the hierarchical medical system in China, the capitation payment standards calculated using municipal-level medical insurance data rather than that from Primary Medical Institutions will better incentivize Primary Medical Institutions to attract patients.

The data utilized in this study were obtained from the medical insurance settlement records provided by the Healthcare Security Bureau of GZ. This dataset covers all full-year outpatient medical insurance settlement data in GZ for 2023 and 2024 (Table 1), including general outpatient services and the selected OCSDs—hereinafter collectively referred to as *Outpatient Services*.

**Table 1:** Dataset of the study.

**Data name**	**Variable type**	**Connotation**
Insurance type	Categorical	310 = UEBHI 390 = URRBHI
Gender	Categorical	1 = Male 2 = Female
Age	Continuous	
Class A OCSDs	Categorical	0 = Non-Class A OCSDs 1 = Single Class A OCSDs 6 = Two Class A OCSDs 7 = Three or more Class A OCSDs
Inpatient status	Categorical	1 = Hospitalized during the previous year 0 = Non-hospitalized during the previous year
Length of hospital stay	Continuous	Calculated as discharge date minus admission date
Fund expenditure	Continuous	

### 3.2 Methods

Existing research has demonstrated that medical expenditures are influenced by multiple factors, including patients' gender, age, medical insurance enrollment status, and disease severity (5). As noted earlier, a one-size-fits-all capitation payment model may no longer align with the evolving needs of contemporary society; thus, there is a need to establish more reasonable differentiated payment standards. The multiple linear regression model is suitable for calculating capitation payment standards, as it can quantify the independent effects of multiple explanatory variables on a continuous dependent variable while controlling for confounding factors. Specifically, this study will construct a multiple linear regression model with medical insurance fund expenditure (*Y*) as the dependent variable, and core independent variables including gender (*G*), age (*A*), Class A OCSDs status (*P*), inpatient status (*H*), and length of hospital stays (*D*). The model is formulated as follows:


$$\begin{array}{l} Y_{i}=cons+\beta_{0}G_{i}+\beta_{1}A_{i}+\beta_{2}P_{i}+\beta_{3}H_{i}+\beta_{4}D_{i} \end{array}$$

## 4 Research model

### 4.1 Consideration of included factors

Previous studies have explored the inclusion of indicators such as age, gender, and comorbidities in the design of capitation payment standards (25), but these studies were limited to patients with diabetes mellitus. However, no relevant research has been conducted on the broader general population. In China's policy practice, UEBHI and Urban and Rural Resident Basic Healthcare Insurance (URRBHI) typically have distinct outpatient reimbursement limits, reimbursement ratios, and deductibles, among other provisions. Thus, the design of capitation payment standards should first distinguish between UEBHI and URRBHI.

The selection of risk adjustment factors in this study adheres to the dual principles of statistical significance and policy operability, and fully considers the actual data acquisition capacity of China's medical insurance settlement system. Gender, age, Class A OCSDs status, and inpatient status were finally identified as core factors, not only because all four indicators were statistically significant in both UEBHI and URRBHI regression models (*p* = 0.000) and could effectively quantify the risk differences of the insured population, but also because these indicators are objective and standardized transaction data in the medical insurance settlement system that can be directly extracted and have high comparability across regions and time. In contrast, indicators such as socioeconomic status, behavioral risk factors, and detailed comorbidity burden are mostly derived from census surveys, questionnaire investigations, or electronic medical records. The collection of such data faces problems such as high administrative costs, inconsistent statistical standards, and poor data sharing between different departments in China's current medical insurance practice, which makes it difficult to apply them to the large-scale formulation of capitation payment standards. In addition, the hierarchical diagnosis and treatment system and family doctor contract service system implemented in China can form a complementary mechanism with the four core risk factors: through the assessment of primary medical institutions' service quality (e.g., standardized management rate of chronic diseases), the potential provider gaming behavior caused by limited risk factors can be further constrained, and the goal of risk adjustment can be achieved indirectly.

The results of linear regression analysis indicated that four indicators: gender (*p* < 0.001), age (*p* < 0.001), Class A OCSDs status (*p* < 0.001), and inpatient status (*p* < 0.001)—were statistically significant in both the UEBHI and URRBHI regression models (Table 2). In contrast, length of hospital stays was not statistically significant in the UEBHI capitation payment regression model that incorporated all candidate indicators (*p* = 0.917). Based on the premise that a unified region should adopt a consistent set of indicators for capitation payment standard design, gender, age, Class A OCSDs status, and inpatient status were ultimately selected as the key factors for inclusion in the model.

**Table 2:** Model fit and test of influencing factors for UEBHI and URRBHI.

**Insurance type Model**	**UEBHI**				**URRBHI**			
	**1**	**2**	**3**	**4**	**1**	**2**	**3**	**4**
Gender	^***^	^***^	^***^	^***^	^***^	^***^	^***^	^***^
Age	^***^	^***^	^***^	^***^	^***^	^***^	^***^	^***^
Class A OCSDs	^***^	^***^	^***^	^***^	^***^	^***^	^***^	^***^
Inpatient status		^***^		^***^		^***^		^***^
Length of hospital stays			^***^	^***^			^***^	0.0917
*R* ^2^	0.2217	0.2367	0.2229	0.2369	0.1799	0.1859	0.1806	0.1860

^***^Represents *p* < 0.001.

### 4.2 Categories design

#### 4.2.1 Age categories

The division of age groups involves two core issues that need clarification. First, whether the age grouping follows the equal-interval principle, and second, how to set the interval standard for each group. Currently, there are two mainstream grouping paradigms in academic research and practice: equal-interval grouping based on fixed age intervals, and unequal-interval grouping integrating the actual characteristics of the population.

The core advantage of the equal interval grouping method lies in its simplicity of operation and clear calculation logic. However, it may lead to excessive concentration of samples in certain age groups, thereby causing systematic bias in the results of overall medical expenditure calculations. The setting of grouping intervals directly affects the number of groups: excessively narrow intervals can improve the accuracy of expenditure calculations but result in a sharp increase in grouping dimensions, which defeats the purpose of accurately designing capitation payment standards; conversely, excessively wide intervals can streamline the grouping structure and reduce the number of categories but inevitably reduce the precision of standard setting.

**Figure 1:**
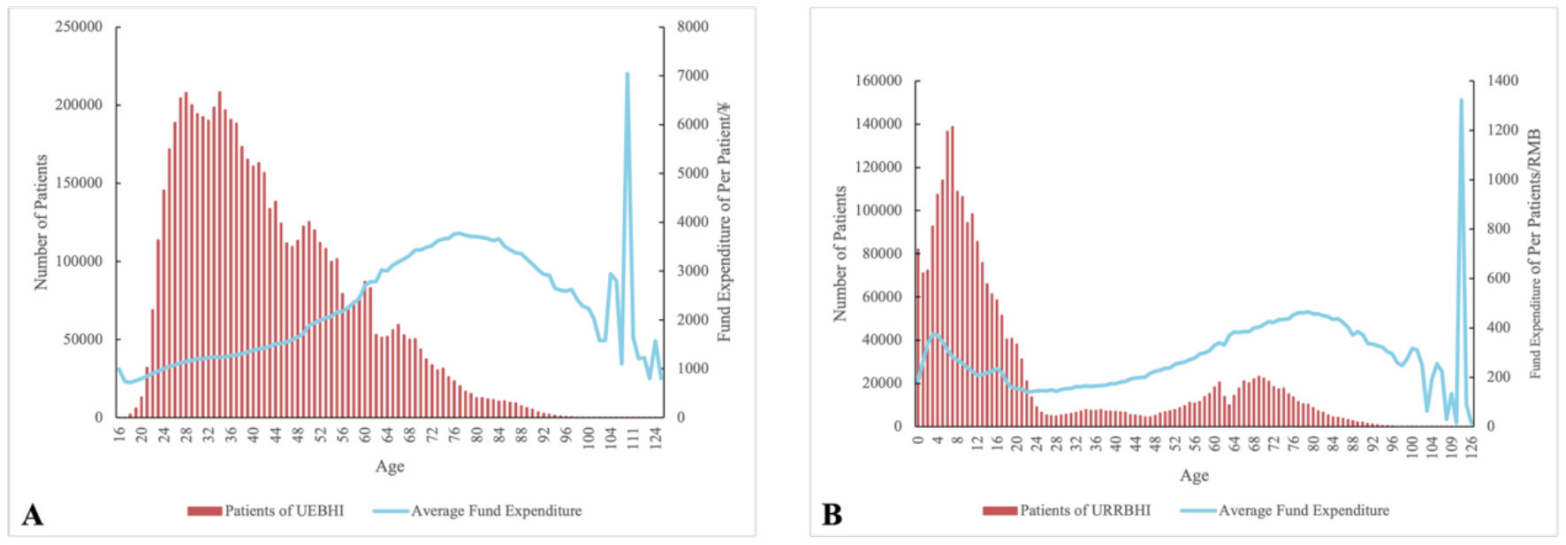
Fund expenditures and by age in the target city. **(A)** UEBHI, **(B)** URRBHI.

Statistical results of the age and medical insurance fund expenditures of UEBHI and URRBHI patients in the sample city in 2024 indicate that the population aged 100 and above has a small sample size. The medical insurance reimbursement expenses corresponding to this age group exhibit sharp fluctuations due to significant impacts from individual patients. Therefore, the age group of 100 years and above was merged into a single group. For the entire population aged 0–100, URRBHI are relatively stable with little fluctuation, but a trend of first increasing and then decreasing can be observed in the 0–5 age group. In contrast, UEBHI show a distinct trend of first rising and then falling among those aged 55–100. Based on these findings, this study divided the age groups of the entire city into the following categories (Figure 1).

As noted earlier, on the basis of age group division, it is necessary to further reduce the number of groups using *K*-means clustering with 2024 medical insurance fund expenditures as the criterion. Before conducting *K*-means clustering for age and disease grouping, the per capita outpatient medical insurance fund expenditure was standardized using the *Z*-score method to eliminate the influence of data scale and magnitude on clustering results.

**Figure 2:**
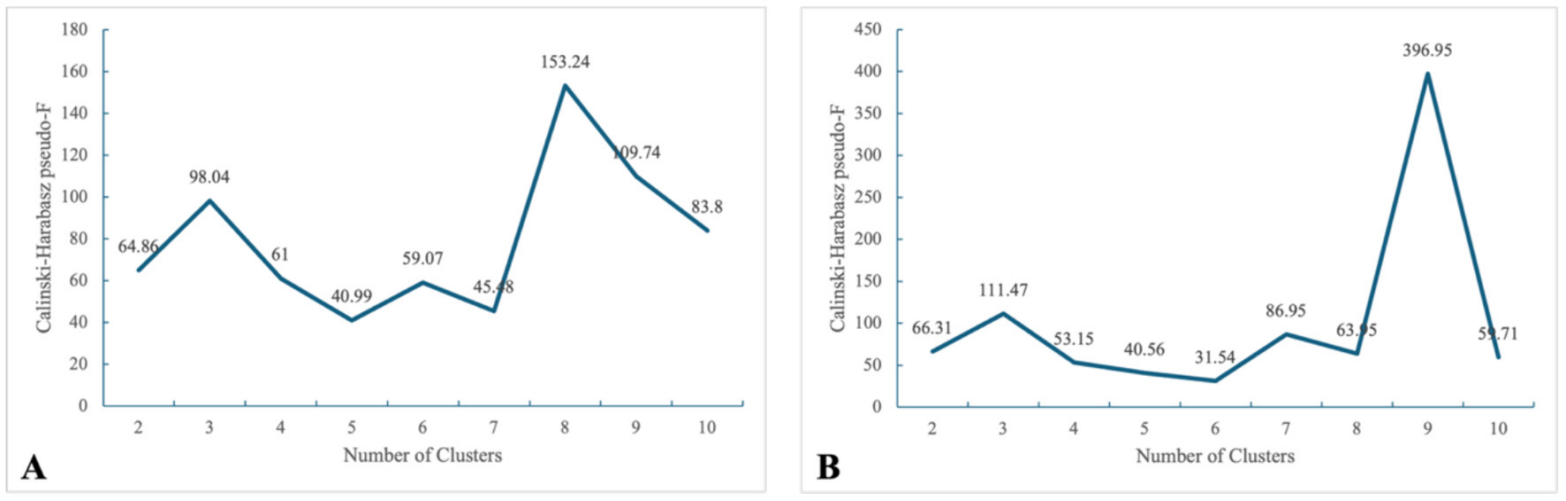
Calinski–Harabasz pseudo-F of different clusters. **(A)** UEBHI, **(B)** URRBHI.

Ultimately, the age groups of UEBHI (Table 3) and URRBHI (Table 4) insured individuals were both clustered into 3 categories, which were verified using the intra-group coefficient of variation (CV) and Calinski–Harabasz index (Figure 2). Although the model yielded higher Calinski–Harabasz indices when the number of clusters exceeded seven, an overly granular age grouping would substantially increase the administrative costs of healthcare insurance administration. Therefore, this study ultimately selected the number of clusters corresponding to the first peak of the Calinski–Harabasz index as the optimal grouping scheme, balancing statistical fit with policy implementability. For the sake of consistency in grouping results, the same age grouping results as those in 2024 were adopted for 2023.

**Table 3:** Age categories of UEBHI.

**Age categories**	**Age range**	**Number**	**Fund expenditure/RMB**	**CV**
Category 1	16–25	554,232	974	0.17
	26–35	1,983,435	1,189	
	36–45	1,595,274	1,380	
Category 2	46–55	1,124,469	1,857	0.18
	56–60	390,111	2,395	
	96–100	1,779	2,517	
	101+	221	1,757	
Category 3	61–65	295,853	2,930	0.10
	66–70	257,012	3,318	
	71–75	159,519	3,585	
	76–80	88,932	3,743	
	81–85	57,683	3,633	
	86–90	38,454	3,335	
	91–95	11,055	2,899	

**Table 4:** Age categories of URRBHI.

**Age categories**	**Age range**	**Number**	**Fund expenditure/RMB**	**CV**
Category 1	11–15	387,636	217	0.17
	16–25	312,191	182	
	26–35	62,240	156	
	36–45	66,643	178	
	46–55	73,010	245	
	101+	86	233	
Category 2	0–5	539,830	318	0.11
	6–10	585,400	275	
	56–60	70,390	305	
	61–65	77,039	360	
	91–95	4,532	329	
	96–100	836	276	
Category 3	66–70	109,514	398	0.06
	71–75	90,174	430	
	76–80	55,224	459	
	81–85	28,474	446	
	86–90	14,346	395	

#### 4.2.2 Diseases categories

In terms of the number of diseases, 83.86% of UEBHI insured patients and 89.62% of URRBHI insured patients in the sample city did not have any risk-adjusted Class A OCSDs. Among the remaining insured patients with risk-adjusted Class A OCSDs, most had a single OCSD. Therefore, it is necessary to reclassify single diseases. For patients with combinations of multiple diseases, a theoretically excessive number of groups would result (276 potential combinations for two diseases and 2024 for three diseases), making this approach unsuitable for grouping. Additionally, due to the relatively small number of such patients, this study divided patients with multiple diseases into two subgroups: those with two diseases and those with three or more diseases.

**Figure 3:**
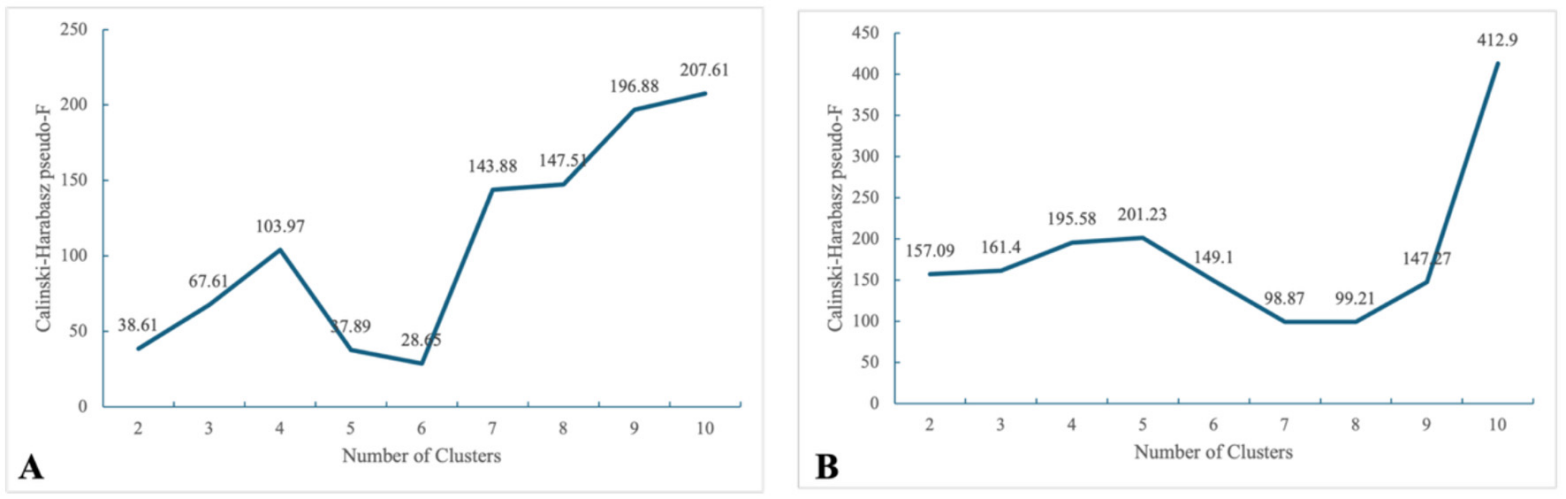
Calinski–Harabasz pseudo-F of different clusters. **(A)** UEBHI, **(B)** URRBHI.

Single diseases were further reduced using *K*-means clustering with medical insurance fund expenditures as the criterion. Ultimately, the single OCSDs of UEBHI (Table 5) and URRBHI (Table 6) insured patients were clustered into 4 and 5 groups, respectively, which were verified using the intra-group CV and Calinski–Harabasz index (Figure 3). For the same considerations as in the age grouping, this study ultimately selected the number of clusters corresponding to the first peak of the Calinski–Harabasz index as the optimal grouping scheme. For the sake of consistency in grouping results, the same disease grouping results as those in 2024 were adopted for 2023.

**Table 5:** Diseases categories of UEBHI.

**Diseases categories**	**Disease**	**Number**	**Fund expenditure of per patient/RMB**	**CV**
Category 1	PD	2,846	5,113	0.07
	OA	3,025	5,038	
	UC	1,264	4,924	
	LC	2,337	4,685	
	CRI–ND	2,935	4,268	
Category 2	SLE	6,711	3,981	0.07
	RA	7,131	3,903	
	Hyperlipidemia	53,957	3,660	
	CG	5,625	3,435	
	SCD	7,501	3,391	
	COPD	7,045	3,337	
	CHD	29,610	3,269	
	BA	9,251	3,217	
Category 3	Epilepsy	4,689	2,971	0.08
	Hypertension	411,616	2,722	
	DM	122,546	2,651	
	CHF	2,712	2,650	
	Hypothyroidism	3,378	2,527	
	CD	993	2,345	
Category 4	AS	4,989	1,902	0.17
	Psoriasis	1,529	1,882	

**Table 6:** Diseases categories of URRBHI.

**Diseases categories**	**Disease**	**Number**	**Fund expenditure of per patient/RMB**	**CV**
Category 1	Epilepsy	2,394	676	0.01
	SLE	767	670	
Category 2	BA	822	567	0.01
	PD	687	566	
Category 3	CRI-ND	618	518	0.04
	CHD	6,154	516	
	RA	857	494	
	UC	74	493	
	CG	384	490	
	SCD	2,475	487	
	DM	29,618	474	
	Hypertension	118,081	468	
	COPD	2,293	464	
Category 4	OA	54	422	0.06
	Hyperlipidemia	3,335	378	
	CHF	1,073	355	
	CD	238	350	
Category 5	LC	466	324	0.08
	Hypothyroidism	249	323	
	Psoriasis	165	287	
	AS	415	276	

#### 4.2.3 Proportional conversion of multi-year calculation standards

Due to their particularity, medical expenditures are prone to the impacts of random fluctuations and specific shocks. Factors such as phased public health events, adjustments to medical service supplementary prices, revisions to the drug catalog, and abnormal changes in medical-seeking behavior may all cause single-year data to deviate from the long-term equilibrium level of medical service costs. Weighted averaging of multi-year data can offset the impacts of accidental deviations through the trend smoothing effect in statistics, thereby making the calculation results closer to the true cost level of medical services.

Meanwhile, by assigning reasonable weights to the 2023 and 2024 data, the 2023 calculation results can maintain the basic stability of the standards and ensure the predictability of medical service supply by medical institutions; the weight assigned to the 2024 calculation results can reflect dynamic cost changes, avoiding the risk of fund over expenditure caused by outdated standards. The formula for the proportional conversion of multi-year calculation standards is as follows, where *S* represents the capitation payment standards calculated in different years, and ω represents the corresponding weights (ω_1_+ω_2_ = 1).


$$\begin{array}{l} S_{2025}=S_{2023}\times \omega_{1}+S_{2024}+\omega_{2} \end{array}$$

The pre-determined capitation payment model inherently faces the problem of patient dumping. therefore, the formulation of capitation payment standards should strike a balance between medical insurance cost control and meeting basic medical service needs.

## 5 Results

### 5.1 Annual capitation payment standards

The capitation payment standards for all population types are the sum of the capitation standards of each subgroup and the intercept. Since males aged 16–45 years, without Class A OCSDs, and non-hospitalized (UEBHI, Table 7) and males aged 11–55 years or over 100 years, without Class A OCSDs, and non-hospitalized (URRBHI, Table 8) were set as the reference groups in the regression model, their capitation payment standards in the corresponding subgroups are all 0 RMB. Thus, the capitation standard for this reference group equals the intercept of the regression model. For all other, the capitation payment standards are calculated by adding their respective subgroup-specific values to the model intercept.

**Table 7:** Annual capitation payment standards of UEBHI.

**Factor**	**Categories**	**S_2023_/RMB**	**S_2024_/RMB**
Gender-Age	1–1	0.00	0.00
	1–2	182.22 ± 4.81	208.86 ± 4.92
	1–3	770.91 ± 6.21	854.26 ± 6.39
	2–1	287.88 ± 3.43	361.58 ± 3.45
	2–2	874.22 ± 4.62	995.47 ± 4.76
	2–3	1,208.81 ± 6.05	1,352.53 ± 6.24
Class A OSCDs	0	0.00	0.00
	1	2,571.35 ± 29.24	2,855.22 ± 30.96
	2	1,428.16 ± 9.87	1,641.07 ± 9.96
	3	831.39 ± 5.13	876.68 ± 5.36
	4	1,304.31 ± 42.33	701.55 ± 42.58
	5	2,025.32 ± 6.95	2,120.24 ± 7.27
	6	3,598.73 ± 11.7	3,731.01 ± 12.38
Inpatient Status	0	0.00	0.00
	1	695.66 ± 4.13	789.63 ± 4.25
_cons		818.54 ± 2.69	919.71 ± 2.67

**Table 8:** Annual capitation payment standards of URRBHI.

**Factor**	**Categories**	**S_2023_/RMB**	**S_2024_/RMB**
Gender-age	1–1	0.00	0.00
	1–2	81.79 ± 0.99	102.94 ± 1.01
	1–3	26.16 ± 1.86	29.08 ± 1.87
	2–1	−2.09 ± 1.09	5.06 ± 1.10
	2–2	61.59 ± 1.01	79.95 ± 1.03
	2–3	21.84 ± 1.59	24.82 ± 1.61
Class A OSCDs	0	0.00	0.00
	1	433.35 ± 9.39	449.14 ± 9.33
	2	350.27 ± 13.61	330.00 ± 13.50
	3	246.70 ± 1.49	232.30 ± 1.52
	4	139.38 ± 8.22	131.81 ± 7.67
	5	107.67 ± 15.37	80.46 ± 14.57
	6	529.04 ± 2.15	519.80 ± 2.16
	7	839.10 ± 4.32	833.87 ± 4.25
Inpatient status	0	0.00	0.00
	1	40.31 ± 1.06	50.67 ± 1.05
_cons		173.57 ± 0.78	184.40 ± 0.79

From the perspective of gender, the capitation payment standards for different genders in the same age group show completely opposite trends between UEBHI and URRBHI: for UEBHI insured individuals, males have higher standards than females, while the opposite is true for URRBHI. From the comparison of results between the 2 years, although the 2023 capitation standards are slightly higher than those in 2024 for a small number of diseases, both UEBHI and URRBHI exhibit an overall trend of higher standards in 2024 than in 2023. This phenomenon is closely related to the improvement of medical service capacity, socioeconomic development, and the expansion of the medical insurance catalog, among other factors.

### 5.2 Municipal-level simulation results in the target city

To investigate the outcomes of the weight design for the proportional conversion of multi-year calculation standards and the development of risk-adjusted outpatient capitation payment standards, a simulation was conducted using the population with actual medical-seeking behavior in 2024. Since both UEBHI and URRBHI exhibit higher capitation standards in 2024 than in 2023, there is a positive correlation between total capitation-based medical insurance fund expenditures and the weight assigned to 2024 for both insurance schemes.

**Figure 4:**
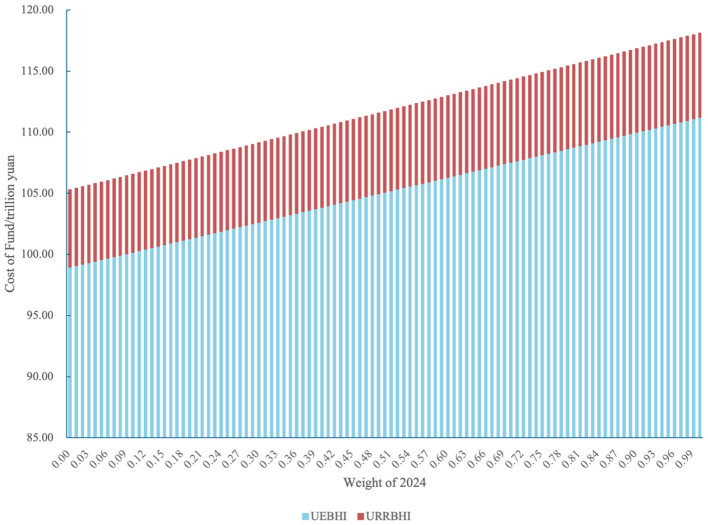
Simulated total capitation payment for UEBHI and URRBHI under different weights.

As shown in the simulation results (Figure 4), under extreme scenarios (with the weight for 2024 set to 0 and 1, respectively): (1) For UEBHI, compared to the actual 2024 expenditure of 11.121 billion RMB, the model achieves savings of 1.226 billion RMB and a deficit of 69.46 RMB, respectively. (2) For URRBHI, relative to the actual 2024 expenditure of 0.69 billion RMB, the model yields savings of 0.057 billion RMB and 84.94 RMB, respectively. The total municipal-level medical insurance fund savings range from 20.48 RMB to 1.283 billion RMB. When both years are assigned equal weights (ω_1_ = ω_2_ = 0.50), UEBHI achieves savings of 0.613 billion RMB and URRBHI achieves savings of 0.028 billion RMB. The aggregate municipal savings amount to 0.641 billion RMB, accounting for 5.47% of the total actual 2024 expenditures (calculated as 0.641/11.811, where 11.811 billion RMB is the sum of UEBHI and URRBHI actual expenditures).

## 6 Discussion

This study takes an economically developed city in southern China as a case study, constructing a risk-adjusted outpatient capitation payment model incorporating gender, age, Class A OCSDs, and inpatient status. The feasibility of the model was validated using medical insurance settlement data from 2023 to 2024.

### 6.1 Interpretation of core research results and policy relevance

#### 6.1.1 Validation of the effectiveness of risk adjustment factors

Through multiple linear regression, this study confirmed that gender, age, Class A OCSDs status, and inpatient status had significant impacts on the fund expenditures of both UEBHI and URRBHI (*p* < 0.001). However, the length of hospital stay was not statistically significant in the UEBHI model (*p* = 0.917). Ultimately, the above four indicators were identified as core risk adjustment factors. This result aligns with previous domestic studies—in Tianjin's capitation payment reform for diabetes, age and complications (especially diabetes-related complications) were proven effective in distinguishing patients' risk levels (25). This study further expands the applicable population from a single disease group to the general population and adds the inpatient status indicator, addressing the deficiency that traditional capitation payment fails to account for fluctuations in patients' illness severity within a year.

From the perspective of grouping results, the clustered grouping of age and diseases effectively balances accuracy and operability. Taking UEBHI as an example, age was divided into 3 categories via *K*-means clustering, with the lowest intra-group coefficient of variation (CV) being only 0.10, indicating small differences in fund expenditures among patients within the same group. Class A OCSDs were clustered into 4 categories based on fund expenditure levels. Among them, diseases such as Parkinson's Disease (PD) and Osteoarthritis (OA) were classified into the highest-risk group due to their per capita fund expenditure exceeding 4,200 RMB, while Ankylosing Spondylitis (AS) and Psoriasis were placed in the lowest-risk group due to relatively lower expenditures. This grouping method is highly consistent with the differences in clinical treatment costs. It not only avoids the issue of medical institutions selectively admitting patients caused by “one-size-fits-all” payment but also does not increase the management costs of healthcare insurance agencies due to overly detailed grouping, which is in line with the reform requirement of “standardizing basic outpatient payment units” in the *14th Five-Year Plan for National Healthcare Security*.

#### 6.1.2 Impact of healthcare insurance type differences on payment standards

The study found that UEBHI and URRBHI showed significant differences in the gender-based variation and absolute value level of payment standards. In terms of gender, UEBHI had higher payment standards for males than females (e.g., 46–55 age group in 2024: 1,857 RMB for males vs. 995.47 RMB for females), while the opposite was true for URRBHI (e.g., 66–70 age group in 2024: 430 RMB for females vs. 398 RMB for males). In terms of absolute values, the overall payment standards of UEBHI (with a maximum per capita of 3,743 RMB in 2024) were much higher than those of URRBHI (with a maximum per capita of 459 RMB in 2024).

This result suggests that the reform of outpatient capitation payment must fully consider the system stickiness of healthcare insurance and cannot adopt a unified standard. In the previous outpatient capitation payment pilot in Jinhua, Zhejiang, the failure to distinguish between insurance types led to insufficient reimbursement for URRBHI patients and waste of UEBHI funds (7). This study provides a technical solution to this problem by designing payment standards separately for different insurance types, which is also consistent with the requirement of “optimizing outpatient security policies by category” in the *Guiding Opinions on Establishing and Improving the Outpatient Mutual-Aid Mechanism for Employee Basic Medical Insurance* (2021).

#### 6.1.3 Fund-saving effect of multi-year weight design

To address the issue that single-year data may be disturbed by accidental factors such as public health events and adjustments to the drug catalog, this study introduced a weighted average mechanism using data from 2023 to 2024. Under the scenario where the weight was 0.5, UEBHI saved 613 million RMB, URRBHI saved 28 million RMB, with a total savings of 641 million RMB, accounting for 5.47% of the actual total expenditure in 2024. If only 2023 data were used (with a weight of 1.0), UEBHI could save 1.226 billion RMB, but the excessively low standard might lead medical institutions to turn away patients with severe illnesses. If only 2024 data were used (with a weight of 1.0), UEBHI would face a deficit of 69.46 RMB.

In practice, this weight design balances the dual goals of cost control and service guarantee. Unlike China Taiwan Region's “capitation payment under global budgeting”, which only focuses on total expenditure control (22), this study smooths trends through multi-year data. It not only prevents medical institutions from adjusting their service behaviors due to short-term standard fluctuations but also ensures that fund expenditures are aligned with the rising trend of medical costs, providing technical support for the sustainability of healthcare insurance funds.

### 6.2 Comparison with existing research and theoretical supplements

Previous studies mostly focused on a single disease or a single type of healthcare insurance. In contrast, this study covers the general population and two types of healthcare insurance, and for the first time incorporates “inpatient status” into risk adjustment factors. It confirms that patients with a history of hospitalization within a year require a higher payment standard (789.63 RMB for UEBHI in 2024), providing a new idea for solving the problem of “poor connection between inpatient and outpatient services”. By differentiating capitation payment standards, medical institutions are guided to focus on the full-cycle health management of patients rather than just the outpatient phase.

Compared with the APG payment model in Jinhua, Zhejiang, the risk-adjusted capitation payment in this study has a lower implementation threshold. APG relies on high-quality outpatient diagnosis data and complex grouping algorithms, while the core factors of this study (gender, age, OCSDs status, and inpatient status) can be directly obtained from the healthcare insurance settlement system without additional collection of clinical data. This makes it more suitable for promotion in regions with unbalanced medical informatization levels. In addition, APG is prone to moral hazards such as “splitting visits” to obtain unreasonable insurance reimbursements. In contrast, this study adopts “capitation payment by person”, which fundamentally curbs the motivation of medical institutions to induce patients to make multiple visits.

In international comparisons, this study also demonstrates local characteristics. The mixed payment model (capitation plus FFS) in Ontario, Canada, reduced the volume of primary care services but failed to consider the special needs of patients with chronic diseases (19). In contrast, this study incorporates high-incidence chronic diseases such as hypertension and diabetes into key guarantees through the grouping of Class A OCSDs. This not only conforms to China's medical reform direction of prioritizing primary care but also specifically addresses the heavy outpatient burden of patients with chronic diseases, providing a Chinese solution for the reform of outpatient payment methods in developing countries.

### 6.3 Practical implications and policy recommendations

#### 6.3.1 Providing an operable technical solution for outpatient capitation payment reform

The technical framework of “risk factor screening—grouping clustering—multi-year weight adjustment” constructed in this study can be directly referenced by other regions. Specifically, in terms of risk factor selection, it is recommended to prioritize indicators that are easily accessible in the healthcare insurance settlement system (such as age, disease diagnosis, and hospitalization history) to reduce reliance on clinical data. In terms of grouping design, K-means clustering can be used to balance accuracy and management costs. In terms of multi-year weight setting, dynamic adjustments should be made according to the local fund growth rate. For years with a high growth rate (e.g., UEBHI growth rate of 80.58% in 2023 in this study), the weight can be appropriately reduced to avoid waste caused by excessively high standards.

#### 6.3.2 Promoting the coordination of healthcare insurance and medical service system reform

The results of this study indicate that the reform of outpatient capitation payment needs to be combined with family doctor contract services and the hierarchical diagnosis and treatment system. Guangzhou has previously linked capitation payment with family doctor contracts. This study further confirms that by adjusting risks to increase the payment standards of primary medical institutions for admitting patients with chronic diseases (e.g., the payment standard for patients with Class A OCSDs is higher than that for ordinary patients), patients can be guided to seek initial treatment at primary care institutions, promoting the implementation of hierarchical diagnosis and treatment. It is recommended that in the future, the quality of family doctor contracts (such as the standardized management rate of chronic diseases) be incorporated into the adjustment factors of payment standards to form a closed-loop incentive of “payment—service—quality”.

### 6.4 Research limitations

This study has several limitations that need to be acknowledged. First, the research is a single-case study based on an economically developed city in southern China, and the conclusions have certain context-specific characteristics rather than national generalizability. China's vast territory leads to pronounced regional variation in healthcare resource allocation, urban-rural medical service capacity, residents' disease spectrum, and medical insurance fund strength (26). GZ, as an economically developed city, has the advantages of sufficient medical insurance funds, high-quality medical resources, and a relatively complete medical insurance settlement system, which is quite different from less-developed regions with insufficient fund support and weak primary medical care capacity. Therefore, the risk-adjusted capitation payment framework constructed in this study cannot be directly copied to other regions, especially less-resourced areas.

Second, this study has certain limitations in the selection of risk adjustment factors. The four core factors selected in this study are relatively simple compared with contemporary international risk-adjustment frameworks. Indicators such as socioeconomic status, behavioral risk factors (smoking, drinking, exercise), detailed comorbidity burden, prior-year medical expenditure, and geographic variation are not included in the model, which to a certain extent limits the explanatory capacity of the regression model and may not be able to capture the more refined risk differences of the insured population. The main reason for the absence of these variables is the limitation of data sources: the research data is only from the medical insurance settlement system, and there is no effective linkage with census data, electronic medical record data, and health survey data. In future research, we will try to build a multi-source data fusion platform by linking with the local statistical bureau, medical institutions, and health administrative departments, incorporate the above-mentioned refined indicators into the risk adjustment framework, and further improve the accuracy of the model in distinguishing population risk differences. At the same time, we will also explore the application of machine learning algorithms in risk-adjustment to make up for the deficiency of traditional linear regression models in capturing non-linear relationships between variables.

## 7 Conclusion

This study built a risk-adjusted outpatient capitation model for China's UEBHI and URRBHI using GZ's 2023–2024 data, with 4 key factors. It optimized groupings via clustering, used multi-year weights to save funds (641 million RMB at equal weights), and revealed insurance type differences. It offers a referenceable framework but has sample limits, with future scope for refining factors.

## Data Availability

The original contributions presented in the study are included in the article/supplementary material, further inquiries can be directed to the corresponding authors.
